# Ineffective control of Epstein–Barr virus infection is seen in MS: What is next?

**DOI:** 10.1002/ctm2.1596

**Published:** 2024-02-22

**Authors:** Paulus Rommer, Elisabeth Puchhammer‐Stöckl, Hans Lassmann, Thomas Berger, Hannes Vietzen

**Affiliations:** ^1^ Department of Neurology Medical University of Vienna Vienna Austria; ^2^ Comprehensive Center for Clinical Neurosciences and Mental Health Medical University of Vienna Vienna Austria; ^3^ Center for Virology Medical University of Vienna Vienna Austria; ^4^ Center for Brain Research Medical University of Vienna Vienna Austria

The cause of multiple sclerosis (MS) is not known. Incidence and prevalence rates differ in the various regions of the world.^1^ Migration studies have shown that age at migration has a significant influence on whether the risk of developing MS corresponds to the country of origin or the host country.^2^ Differences in the genetic background in the population^3^ alone does not seem to be sufficient to explain the differences in MS prevalence. Disease susceptibility is due to a complex interaction between genetic predispositions and environmental factors, including infections. And indeed for decades, MS has been associated with preceding Epstein–Barr virus (EBV) infections, and a recent epidemiological study suggests that primary EBV infection during adolescence is an essential trigger of the disease.^4^ However, the question remains as to why so many people carry EBV but only a small fraction develop MS. Furthermore, it is unclear, how EBV infection triggers or drives the disease process.^5^ Persistent infection and repeated virus reactivation may serve as a stimulus for chronic inflammation within and outside the nervous system, either directly or by creating a persistent pool of pro‐inflammatory B lymphocytes.^6^ Alternatively, autoimmune responses may be induced through molecular mimicry of antigens shared between EBV proteins and antigens of the central nervous system (CNS), as described for glial cell adhesion molecule (GlialCAM),^7^ αB‐crystallin,^8^ anoctamin,^9^ myelin basic protein, and others.^10^, ^11^, ^12^

For the first time, data are now reported, which provide an explanation for some of these questions.^13^ All MS patients in the study had high GlialCAM_370‐389_‐IgG levels, but also 40% of non‐MS had similarly high levels. This allowed to analyse immunological differences between MS and healthy controls with a high GlialCAM_370‐389_‐directed immune response.

The study revealed that it is primarily the complex interplay of the immune system of the individual that is responsible for the development of MS.

EBV was already earlier supposed to trigger MS, but now it was shown that MS patients were infected mostly with specific EBV strains that encoded for a distinct EBV latent membrane protein 1 (LMP‐1)‐derived peptide. This peptide induced a high expression of the human leukocyte antigen (HLA)‐E, and this in turn led to an increased inhibition of a specific subtype of natural killer (NK) cells: NKG2A^+^ NK cells. Thereby this distinct virus variant, especially when presented by a specific genetic variant of the HLA‐E, contributed significantly to the development of MS.

In addition, it was revealed that NKG2C^+^ and NKG2D^+^ NK cells provided disease protection. Healthy controls with high GlialCAM_370‐389_‐IgG levels were infected with distinct human cytomegalovirus (HCMV) variants that encoded for particular UL40 peptide variants, leading to high levels of protective NKG2C^+^ NK cells. In contrast, MS patients had substantially decreased NKG2C^+^ NK‐cell levels that were induced either by absent HCMV infections, a genetic deletion of the NKG2C‐receptor, or by distinct viral UL40 peptide variants, limiting NK‐cell conferred protection against MS. Besides NK cells, also T‐cell immunity was analysed and it was shown that GlialCAM_370‐389_‐specificCD8^+^ T‐cell responses were due to decreased NK‐cell responses and increased viral NK‐cell evasion, more potent and long lasting in MS patients than in healthy individuals. These results describe a scenario with less efficient control of EBV infection, but in parallel with an increased T‐cell autoimmune response against CNS epitopes. Taken the findings together and in dependence on various constellations, the odds ratio of developing MS was up to 260‐fold increased.

These results raise the question of whether we have come any closer to clarifying the pathogenesis of MS, and whether it is time to break new ground in therapy? Autoimmunity, induced by molecular mimicry, has for long been suggested to play a role in MS pathogenesis,^12^ but convincing evidence for this assumption is so far missing. All epitopes, shared between EBV‐nuclear antigen (EBNA)1 and CNS antigens are dominantly located in the cytosol, and are not expressed on the cell surface. Thus, it has not been shown and it is very unlikely that respective autoantibodies are per se pathogenic. Although T cells directed against such antigens can trigger inflammation in experimental models, so far, all trials of antigen‐specific desensitisation strategies have failed in MS patients. One possible explanation for such a situation is that EBV‐induced autoimmune responses are not directed against a single CNS target. It will be, thus, of critical importance, to investigate whether the dysregulation of autoimmunity is specific for GlialCAM, whether it is seen to a similar extent for all other antigens, cross‐reacting with EBV, or whether it is due to a general EBV‐induced dysregulation of T‐cell responses. The design of future antigen‐specific desensitisation strategies in MS patients critically depends upon this information. A first candidate to test is αB‐crystallin, due to its high expression in the lesions and the profound T‐cell response in MS patients.^14^

It also remains unclear to what extent EBV will ‘only’ initiate MS, or also induce disease activity. Immune reactions of CD8^+^ T cells against EBV epitopes were shown post mortem in the brains of MS patients,^15^ but still, the questions remains, whether EBV or CNS epitopes trigger the immune reactivity. If EBV infection remains in the CNS as a permanent trigger for disease activity, therapies should target EBV. Indeed, first studies have been reported and launched,^16^, ^17^ but a respective clinical trial did not meet the specific endpoints.^18^ If EBV causes a sustained immune response, it should also be investigated to what extent EBNA1 levels themselves can be used as monitoring or even disease markers. On the other hand, if EBV is not a permanent trigger, the immune response persists due to clonality and memory cells. In this case, not targeting EBV but prevention from EBV infection is needed, and so far our approved treatments aim to decrease this secondary immunoreaction and have proven efficacy.^19^

However, if the connection between EBV and MS is confirmed in follow‐up studies and a risk stratification after EBV infection can be established, it is plausible to intervene with the pathogenesis of MS itself.^5^

To prevent MS, vaccination against EBV in early childhood has been suggested. Even when successful, a result of such a strategy will be seen only after decades from now. More promising could be to try, whether infectious mononucleosis can be prevented, when EBV negative adolescents are immunised. As the development of infectious mononucleosis is an established risk factor,^20^ it is likely that this also reduces the development of MS in the respective population. However, EBV vaccination may also carry the risk of the induction of autoimmunity in patients, susceptible to develop MS. Thus, such vaccination trials have to be carefully monitored, and the study by Vietzen et al.^13^ suggested the immunological and virological parameters, which have to be addressed.

Alternatively, in subjects, who have already acquired EBV, the cytotoxic NKG2C^+^ and NKG2D^+^ NK‐cell response could be boosted. This option appears to be more feasible for vaccination trials in patients with MS, as the target group can be recognised after EBV infection and risk stratification.

Finally, a key aspect of our study is that it sheds new light on the complex interaction between viruses (in this case EBV) and autoimmunity. This could play a role not only for MS, but also for other post‐viral diseases.

## AUTHOR CONTRIBUTIONS

Conceptualization: Paulus Rommer, Elisabeth Puchhammer‐Stöckl, Hans Lassmann, Thomas Berger, Hannes Vietzen; writing—original draft: Paulus Rommer; writing—review & editing: Paulus Rommer, Elisabeth Puchhammer‐Stöckl, Hans Lassmann, Thomas Berger, Hannes Vietzen.

## CONFLICTS OF INTEREST STATEMENT

The authors declare no conflict of interests.

## ETHICS STATEMENT

This article does not contain any studies involving human participants performed by any of the authors.
